# Towards a systems approach for chronic diseases, based on health state modeling

**DOI:** 10.12688/f1000research.11085.1

**Published:** 2017-03-23

**Authors:** Michael Rebhan

**Affiliations:** 1 Novartis Institutes for Biomedical Research, Basel, 4056, Switzerland

**Keywords:** chronic diseases, systems approach, Precision Medicine, computational modeling, disease progression, Markov health state models, Regenerative Medicine, Open Science.

## Abstract

Rising pressure from chronic diseases means that we need to learn how to deal with challenges at a different level, including the use of *systems approaches* that better connect across fragments, such as disciplines, stakeholders, institutions, and technologies. By learning from progress in leading areas of health innovation (including oncology and AIDS), as well as complementary indications (Alzheimer’s disease), I try to extract the most enabling innovation paradigms, and discuss their extension to additional areas of application within a *systems approach*. To facilitate such work, a Precision, P4 or Systems Medicine platform is proposed, which is centered on the representation of *health states* that enable the definition of time in the vision to provide *the right intervention for the right patient at the right time and dose. *Modeling of such *health states* should allow iterative optimization, as longitudinal human data accumulate. This platform is designed to facilitate the discovery of links between opportunities related to a) the modernization of diagnosis, including the increased use of omics profiling, b) patient-centric approaches enabled by *technology convergence*, including *digital health* and connected devices, c) increasing understanding of the pathobiological, clinical and health economic aspects of disease progression stages, d) design of new interventions, including therapies as well as preventive measures, including sequential intervention approaches. Probabilistic *Markov models* of health states, e.g. those used for health economic analysis, are discussed as a simple starting point for the platform. A path towards extension into other indications, data types and uses is discussed, with a focus on *regenerative medicine* and relevant pathobiology.

## Rising pressure from chronic diseases

One of the main challenges our healthcare and biomedical research and development systems are facing, in the age of digitalization and aging populations, is a rising burden from chronic conditions. This burden has a multitude of effects not only on the *Quality of Life* (QoL) and well-being of the patients and their immediate social networks (e.g. family members), but it also triggers increasing discussion about sustainability problems in health-related systems, including the economics of healthcare systems. Medical conditions are defined as being ‘chronic’ when they last 12 months or more, result in functional limitations (which tend to reduce QoL) and/or the need for ongoing medical care (i.e. healthcare resource utilization). Costs associated with chronic conditions are on the rise in many countries, and have been identified as a main driver of medical cost explosion, leading into the economic sustainability discussion. By now, they cause the majority of all healthcare costs in developed countries, with fast-rising prevalence in some emerging countries as well, as their societies increasingly imitate developed countries, including lifestyle, economy and burden from chronic diseases.

For example, in the US, a country that is among the most advanced in terms of this development, 31.5% of the population in 2010 was affected not only by a single, but multiple chronic conditions (MCC), binding more than 70% of all healthcare spending (not considering other costs, outside healthcare budgets, such as social care) ( Gerteis *et al.*, 2014). Chronic diseases overall, including patients with a single chronic condition, account for a vast majority (86%) of healthcare spending in the US ( Gerteis *et al.*, 2014), leading to intensive discussion on how long society can afford to pay for rising healthcare budgets ( Callahan, 2013), which are based on economic models that are largely disconnected from outcomes achieved (EFPIA, 2015). In terms of indications, metabolic (e.g. diabetes), cardiovascular (e.g. heart disease), respiratory (e.g. COPD and asthma), autoimmune (e.g. rheumatoid arthritis), and neurological conditions (e.g. Alzheimer’s, and Parkinson’s disease) are typically among the most commonly observed, depending on the country and population ( Callahan, 2013; Gerteis *et al.*, 2014; Nugent, 2008; and Kvedar *et al.*, 2016; see also the *Global Burden of Disease* study below).

This increase in chronic diseases in both developed and also emerging countries ( Nugent, 2008) represents a challenge that forces us to go back to the design board, in terms of the health-related systems we have created, to increase their ability to cope with what’s growing in terms of challenges. As a recent article that explains the need for such a fundamental redesign puts it: we face a “critical turning point, requiring not only improved health care systems but also a new model of medicine at its foundation” ( Callahan, 2013). Similar statements can be found in the discourse of other disciplines involved in health innovation, including biomedical research and its translation ( Butler, 2008; Cooksey, 2006; Lazebnik, 2002; Munos, 2010; Munos, 2016; Poste, 2011). At the same time, due to medical progress in specific areas, some of the diseases that were almost impossible to survive a while ago, now turn into new types of chronic conditions, e.g. AIDS (where personalized combination therapies have enabled impressive improvements of patient outcomes in a relatively short time, see below). Such new chronic conditions created by medical progress also require sustained care and resources over many years, further increasing chronic disease burden. This trend of medical innovation creating new chronic conditions is likely to continue. “It is now possible, and not uncommon, for someone to have cancer pushed into remission at 65, to persist with well-managed heart disease at 75, and then to acquire Alzheimer’s at 85” ( Callahan, 2013). Therefore, the rise in life expectancy that follows increasing development according to the Western model of modernization of the last 2–3 centuries is accompanied by more time spent in a managed chronic condition. This, in turn, leads to a lively debate on the need to push innovation for ‘healthy aging’, considering not only how *long* we live, but also the QoL of those added years. In that context, what can we learn about ‘healthy aging’, in the absence of a heavy burden from chronic diseases, in populations that do better than average?

## Islands of healthy aging

Comparisons between different human populations (e.g. in different geographies, or between subpopulations that live in the same geographic area) can reveal interesting patterns related to this debate. Studies of human populations that enjoy both a long *and* healthy life compared to others in their proximity (i.e. “islands of healthy aging”), so far have revealed that there are candidate contributing factors for healthy aging at many levels, including genetics, various aspects of lifestyle, environmental context, sociology and culture, and of course economic factors. However, it is important to be cautious about accepting simplified conclusions from such studies, as they suffer from the same fundamental problems as other types of studies in complex human populations, including the risk of unintentionally comparing apples and oranges (which can reveal the wrong factors as being significant), as well as the temptation of jumping from correlations to statements on causation (as it often happens in the mass media, which adds to widespread confusion on the topic).

In the case of the so-called ‘ Blue Zone’ populations of central Sardinia ( Pes *et al.*, 2013), a Mediterranean island with pleasant climatic conditions, various studies aim to identify significant differences between the healthy aging ‘Blue Zone’ populations, which are known as some of the most long-living populations in the Western world, and other Sardinian populations that have a close-to-average life expectancy and health profile during aging. Note that the ‘Blue Zone’ populations in the center of the island are known to have been slower in adopting a modern lifestyle, compared to the people on more accessible coastal areas (a pattern that can be observed in similar landscapes, where accessibility of geographies influences speed of modernization). A statistical analysis of factors that clearly distinguish both Sardinian populations from each other (i.e. Blue Zone populations from the others) revealed occupational aspects (with communities rich in shepherds being healthier than those with more farmers and fishermen), landscape (mountainous terrain being a healthier environment compared to coastal lowlands), and dietary factors (with Barley production associated with healthy aging) as significant. A possible conclusion from such an analysis of correlations could be that healthy aging populations are rather found in areas with many shepherds, who used to spend much time roaming sparsely populated, mountainous areas, and less than in areas with more intensive spurts of activity typical for farmers and fishermen. Other conclusions may be valid as well, and it can be difficult to choose among the alternative conclusions, to inform action.

Based on our current knowledge about the characteristics of healthy aging populations, and risk factors for increased burden from chronic diseases (e.g. from the Framingham and similar longitudinal observational cohort studies; Mahmood *et al.*, 2014), initiatives aimed at reducing chronic disease burden in public health have tried to develop solutions that work in an efficient manner at population level, including educational, political, regulatory and medical initiatives. One of the most visible exemplars for successful paradigms in public health is the reduction of burden associated with smoking and second-hand smoking reduction, highlighting the power of coordinated, interdisciplinary collaboration towards a higher-level health goal. In that context it is important though to point out that much of the evidence we have is, as stated above, only correlative in nature, and that its efficient reduction to practice in terms of the best (combination and/or sequence) of interventions in different populations and settings is anything but trivial (see also Carter, 2015, and the discussion on AIDS and platform applications below). For example, let’s assume that many studies confirm that a shepherd-like lifestyle in mountains with mild climate, regular siesta and associated diet is indeed the one that gives us the most healthy aging experience, how do we extend such a ‘successful lifestyle paradigm’ into another setting that is less peaceful and traditional, e.g. a busy, modern, urban environment with its strong selective pressures on lifestyle and culture?

## Systems approaches

With all the (somewhat fragmented) knowledge we have accumulated, and made increasingly accessible with digitalization, I propose that it is a good time to learn how to “put the pieces of the puzzle together”, by learning how to best link and extend the most successful paradigms. Learning, in this case, means to understand the most powerful combinations of paradigms, where a paradigm can and where it cannot be applied (its ‘domain of validity’), and what adjustments to its implementation are needed to fit a particular situation. Several examples are provided below, e.g. the lessons learned from the modernization of diagnosis in oncology and AIDS, combined with innovation on more personalized (combinatorial) interventions. To achieve this, we need to learn how to better connect relevant ‘pieces of knowledge’ and stakeholders, across disciplines, institutions and other real life barriers, towards increased speed and effectiveness of distributed learning, at systems and community level. This should put following generations into a better position for managing not only problems related to sustainability in health, which our generation is still struggling with, but also problems in other (connected) areas that pose similar challenges.

However, if we do a reality check, of our status quo, this is the type of challenge we, at our current stage of human cultural evolution, have provided only limited evidence so far for actually being able to cope with. Several thousand years after a series of cultural transitions from small communities of hunter-gatherers (with a more limited control over their environment) into increasingly large and complex, globally connected societies (with more widespread effects in our environment, including the most remote corners of our planet), the question poses itself: what is the next stage in our cultural evolution, as a species? Will it actually be possible to overcome obstacles on the path towards multi-stakeholder co-design of healthier and more sustainable systems, and how long will it take?

 In the life sciences, including fields related to medicine and biology, we can find many good initiatives that point into this direction, but also a widespread disbelief among leaders in those disciplines that we will be able to fundamentally change things, because “things that never change” (which translates into the implicit belief that we have reached the end of human cultural evolution, in terms of our ability to manage certain types of complexity, as a human population) and the special characteristics (complexity) of living systems compared to engineered systems (e.g. see Lazebnik, 2002). Good introductions into those important discourses, in the above context, are provided by

- Altman, 2012 (linking the molecular and clinical worlds; role of systems medicine)
- Auffray *et al.*, 2016 (focus on European initiatives, and the need to connect those)
- Barker, 2011 (sustainability of healthcare systems, with US and UK focus)
- Butler, 2008 (the ‘valley of death’ problem, translating innovation to impact)
- Callahan, 2013 (sustainability of medicine, healthy aging, chronic diseases)
- Carter, 2015 (healthy aging, medical philosophy, and public health policies)
- Goodwin, 1999 (evolution of science, from control to participation)
- Koelsch *et al.*, 2013 (economic sustainability of personalized health model in Oncology)
- Lazebnik, 2002 (blind spots in biomedical research, lack of common language)
- Mathews & Pronovost, 2011 (need for better systems integration in medicine)
- Munos, 2010 (from a non-sharing, competitive culture to open science in biomedical R&D)
- Munos, 2016 (innovation crisis in pharma R&D, and economic sustainability of the industry)
- Poste, 2011 (problems related to biomarkers and diagnostics)
- Powell, 2004 (systems approaches in biology, key problems and some trends)
- Pritchard *et al.*, 2017 (translating PM into regular clinical care, key challenges, adoption)

**Figure 1.**
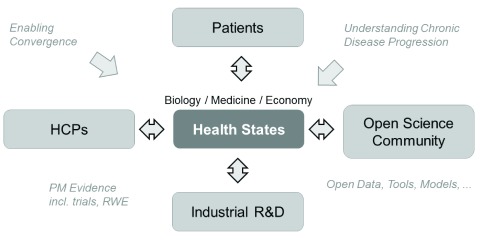
The proposed innovation ecosystem for chronic diseases, with a new platform that engages different health innovation stakeholders, and allows the emergence of interdisciplinary understanding of health states across biology, medicine and health economics in its digital center. The design is based on the ambition that all stakeholders should benefit from the development of this digital center. RWE = real world evidence.

The ability to make progress here requires an increased capability for understanding how different aspects in relevant *subsystems* influence each other dynamically. Recently, we can observe early signs of a transition to a ‘new health innovation ecosystem’ with changes in many subsystems, based on changing roles (e.g. of patients, physicians and pharmacists), processes, habits, and underlying economic models ( Barker, 2011; Beckmann & Lew, 2016; Koelsch *et al.*, 2013; Munos, 2010), as a first symptom of efforts to increase systemic sustainability, as well as the effects of technology advances (see below). The types of challenges we need to tackle includes the need for a discourse of important trade-offs that require careful balancing of the perspectives of multiple stakeholders. For example, as we develop therapeutic solutions for increasingly smaller but more molecularly defined populations in oncology, based on diagnostic modernization (see below), this results in tension between high prizes for targeted therapies and the enormous investment required to develop new targeted therapies in a highly regulated, and cost-intensive industry ( Koelsch *et al.*, 2013; Kostic & Phillips, 2015?). Another fundamental trade-off situation with many consequences for a variety of stakeholders can be found when considering the resources currently involved in the last 5–10 years of life, including elderly, health, social and other care (e.g. by family members). Is a move towards more robots taking care of our elderly the only solution we can imagine, since there will not be enough people around to provide more human versions of care? Resolving such and other interconnected, multi-stakeholder challenges with trade-off tensions in their core will be an important set of problems to address, see Figure 1. In this context, a renewed and more widespread interest in *systems approaches* as a tool for managing such complexity is on the cards. For a brief overview of potentially relevant fields, concepts and tools related to *systems approaches*, see Box 1.

Box 1. **Expertise and tools related to systems approaches**

In this article, I define *systems approaches* as efforts aimed at ‘connecting the pieces of the puzzle’, i.e. a set of connected parts or subsystems (i.e. system components) that influence each other, with an emphasis on understanding the interactions between those parts, and how they contribute to system-level properties. System-level properties include *emergence*, which means that the system displays behaviors that depend on the way how the system components interact with each other, and *robustness*, a property that captures the ability of a system to deal with changes in its environment (e.g. living systems have evolved a collection of complementary system motifs that enhance their ability to cope flexibly with changes of food supply). Systems approaches can build on knowledge and tools from a range of fields, such as systems science, complexity theory, computational modeling of complex natural systems (e.g. in ecology and economy), nonlinear systems theory, self-organizing systems, chaos theory, cybernetics, whole systems thinking, general systems theory, and game theory. Introductory texts into some of the most relevant fields, their key concepts and tools, can be found in Goodwin (1999); Hammond (2005); Lazebnik (2002); Powell (2004); Sterling (2003); and Bousquet *et al.* (2011).

For most of the 19 ^th^ and 20 ^th^ century, our mindset was preoccupied with certain ideas of ‘development’ and ‘civilization’, with mostly negative views of other lifestyles found in ‘less developed’ areas, and a belief in a core role of new technologies to enable even more development towards an even better civilization. As such development, spreading globally, led to increasing awareness about the “other side of the coin”, i.e. negative consequences for human and non-human species, this fueled excitement on finding better ways to understand complex systems that involve living species, e.g. how effects related to human development (e.g. pollution, change of environments, increasing density of human populations, waste) affected the health of ecosystems (e.g. lakes undergoing *eutrophication* based on system shifts, with deadly consequences for the species that used to inhabit this biosphere; Yang *et al.*, 2008). Over time, such ‘ecosystem’-related fields developed the capability to understand recurring principles in that complexity, including the role of the connectivity between individual system components (Sterling, 2002). Interestingly, this revealed common patterns found in many complex systems, adding further fuel to the interest in *systems approaches* as a tool for managing complexity.

The ability to perform experiments, and the use of increasing computational power to develop better *in silico* models, at systems level, played an important role in this process. However, an early attempt to apply developments in those areas to molecular networks involved in disease, in the form of ‘systems biology’ ( Lazebnik, 2002; Powell, 2004; Spivey, 2004), got slowed down by a few fundamental challenges. The effort and time needed to advance our understanding of all relevant system components and their interactions in human health, at sufficient detail for determining the best intervention (i.e. ‘target’) for promoting a transition to a particular health state, is immense, and there is doubt if that vision can even be achieved despite technology advances (e.g. omics technologies that can monitor the state of thousands of such molecules in living systems, see below). As a consequence of this ‘cool down’ on systems biology (enabled by omics profiling), many academic researchers in the field have shifted, over the years, to study simpler systems that are more remote from human complexity first, e.g. (populations of) easier-to-study single cell organisms with more simple genomes and behaviors, while applied research and medical innovation in industry largely focuses on other paradigms for generating starting points for their innovation pipelines, e.g. based on the screening of biological systems that model selected aspects of disease (to find starting points for new therapies). Note that institutes designed around a long-term investment into systems biology approaches, such as Lee Hood’s Institute of systems biology in Seattle, have made considerable contributions to the continued discourse on the need for systems approaches in health innovation, and the development of guiding principles for P4 Medicine (e.g. Bousquet *et al.*, 2011; Hood *et al.*, 2014, and below). However, this discourse if by now substantially different from the ambitions of the systems biology wave about 10 years ago, as the community was getting excited about a new ability to ‘know all the parts’ and put the picture together on their interactions.

At the same time, there is increasing recognition that ‘ **biomarkers’** may become important ‘anchors’ in those complex networks, due to the ability to study their links with medical, economic and other non-biological data related to chronic diseases, including connections between diagnosis and intervention (see below). Scientific discussions related to this shift towards biomarkers ( Burns *et al.*, 2013; Poste, 2011) are one of the origins of the proposed platform. They may also serve as useful scientific bridges between key stakeholders ( Figure 1), e.g. between different Intellectual Property/innovation domains such as the OpenScience community (where their efforts add information on the role of biomarkers and biomarker-based health state models) vs. proprietary therapeutic assets in pharmaceutical R&D pipelines (e.g. where mechanism of action biology of those assets connects with such biomarkers, and health state biology). Similar issues may occur at interfaces between patient/consumer-centric solutions (e.g. through digital health), and those deployed in hospitals (i.e. for health care providers, HCPs), see Figure 1, with their different Intellectual Property/innovation domains. Biomarkers, as they contribute to the development to the interdisciplinary understanding of health states across stakeholders, are therefore an important focus of the proposed systems approach.

Of particular interest, from a systems point of view, will be knowledge related to the ability of different kinds of systems to cope with external changes (i.e. system robustness), including pressures outside the normal range of what the system is typically encountering (short time scales), or what it was encountering during its evolution (longer time scales). In a time of complex interactions between changes in various fields related to chronic diseases, we need to understand more about what makes systems robust despite change, and how the forces that drive change, and their effects, are connected. Kuhn’s thoughts on recurring, cyclic patterns in the history of science that he called ‘paradigm shifts’ (Kaiser, 2012), including the accumulation of ‘anomalies’ that are inconsistent with the dominating paradigm(s), may be helpful. More widespread adoption of tools related to *systems approaches*, outside the existing, rather small group of experts, in areas where theory and practice collide for better learning, will be an enabling development for the proposed platform. In that context, it is important to develop a modeling-based learning process in the public domain, on a neutral platform that involves many stakeholders.

## Relationship with Precision, P4 and Systems Medicine

As different aspects of an emerging consensus on how to develop more sustainable health-related systems are discussed in the literature and other media, due to the early stage of the discussion a variety of terms that capture key elements of the transition are used with inconsistent meaning, adding to overall confusion. Some of the terms that try to capture the ambitions of a ‘new health innovation ecosystem’, range from ‘Precision Medicine’, ‘Personalized Health’ to ‘P4 Medicine (P4 because of the four principles, starting with ‘p’: predictive, personalized, preventive, participatory)’ and ‘Systems Medicine’. For a recent overview on this discourse, see Auffray C *et al.*, 2016; Bousquet J *et al.*, 2011; Flores *et al.*, 2013; Hawgood *et al.*, 2015; Hood *et al.*, 2014; Hood & Price, 2014; Kodrič K *et al.*, 2016; Kostic & Phillips, 2016; Scholz, 2015; Wang *et al.*, 2015; and Wilckens T, 2016. Comparison with the guiding principles of evidence-based medicine is provided by Beckmann & Lew, 2016. Going forward, I will use the simplified abbreviation ‘PM’, as it captures at least some of the more commonly used terms (i.e. Precision/ Personalized/ P4 Medicine) in a simple abbreviation, assuming that *systems approaches* are an important tool on the path to the development of sustainable PM-based systems. The proposed platform is designed in a way that can accommodate the early stage of the emerging consensus in PM, and facilitate its maturation.

## Aims of this article

In this article, I aim to make a contribution to this discourse by 1) discussing potentially reusable, successful paradigms from selected areas of medical innovation, 2) leading to guiding principles for designing a platform that enables multi-stakeholder initiatives, centered on a theory of *health states*. In terms of interdisciplinary interfaces, our focus is on connections between medicine, biology and economy, and initially focus on applications related to *regenerative medicine*. Iterative optimization of the proposed reference *health state* models would be fueled by linking opportunities related to a) the modernization of diagnosis, b) ability to capture *health state* profiles using omics, c) patient-centric approaches based on *technology convergence*, d) increasing understanding of the pathobiology, clinical meaning and health economic aspects of disease progression stages, and e) design of new interventions, including therapies as well as preventive measures.

## Successful paradigms, from leading areas of health innovation

Looking across different areas of medicine, we can notice interesting differences, e.g. in sharing culture, commonly applied tools, mindsets and approach, affecting the translation of advances in knowledge into improved patient outcomes, as well as the generation of new advances that fuel further progress. Here, I would like to highlight a number of successful paradigms with an impact on patient outcomes, and their potential relevance in the above discourse, even outside the problem areas in which they were originally developed.

### Modernization of diagnosis, and personalization of therapy

In discussions on the “valley of death” challenge in health innovation ( Butler D, 2008; Wehling, 2009), which concerns the problem of translating scientific and technical advances into impact at the level of patient outcomes, beyond time-limited clinical studies, oncology is often mentioned as an area of medicine in which there has been relatively good progress in terms of such translation into regular practice. In this medical specialty, many advances in our increasing scientific understanding of the molecular basis of disease, and patient heterogeneity, have been translated into solutions that benefit patients with specific tumor profiles. Looking across different areas in Oncology, the most successful paradigms that evolved effectively couple the modernization of diagnostics (i.e. the ability to determine tumor subtype based on its biological profile) with the use of targeted therapies (which have been designed for a specific tumor type, or a set of tumor types, with a characteristic biological profile). This personalized health paradigm emphasizes understanding of patient heterogeneity at the level of biological profiles, because it was possible to link diagnostic capability at the level of tumor-derived DNA with its interpretation in terms of the biology that drives the growth and survival of that type of tumor, resulting in impressive improvements of patient outcomes in many tumors (where both tools converge). However, this paradigm has also raised economic sustainability concerns, as tension increases between stakeholders who a) invest in the development of solutions based on this paradigm, and b) those who need to pay for healthcare of tumor patient populations, which are increasingly segmented, with many segments associated with relatively high costs (see above, and Koelsch *et al.*, 2013).

In this renowned area of medicine, many innovations based on this paradigm have advanced quite far in the innovation translation pipeline, leading to practical solutions for global deployment, reimbursement in different healthcare systems, education of healthcare providers, and integration into regular care processes. Considering the effort that is required for such a level of system-wide change in the real world, those successes are indeed quite impressive, keeping in mind, however, that there are many areas of medical need that remain a considerable challenge in oncology, including the phenomenon of tumor recurrence despite short or mid-term effects of targeted therapies.

If we consider to extend this paradigm to other areas of medicine, we need to take into account that tumors have many characteristics that are fundamentally different from many common chronic diseases, complicating the application of exact copies of the approach in non-Oncology areas, apart from some exceptions, such as diseases with a strong genetic component (which tend to be rare). Therefore, we need to learn how to consider the particular characteristics of a disease, at a diagnostic and therapeutic level, as we extend the oncology paradigm of personalized health to other indications. This challenge, so far, has been hard to crack, triggering a ‘lessons learned’ discourse that can be quite healthy in the context of a possible adoption of the proposed platform. Understanding the very slow progression of many common chronic diseases, from different angles, is part of the scientific challenge, as outlined below.

Another area of medicine that has witnessed much progress in terms of developing a modern approach to the convergence of innovation in diagnosis and therapy is AIDS. Being an infectious disease makes it a case that is quite different from oncology and most chronic diseases, although aspects of the oncology paradigm of targeted therapy and personalization have been re-used here as well. Once the AIDS epidemic was recognized as a major health challenge, relatively fast progress on understanding the key characteristics of viral populations, and the biology of their interactions with host (defense) biology, has enabled the development of highly personalized combination therapy approaches, depending on the DNA level composition of the viral population in that specific patient, at a particular point in time ( Lengauer & Sing, 2006; Lengauer *et al.*, 2014). As in oncology, much of the progress in this area of medicine was catalyzed by technical progress. For example, easier access to relevant omics technology (see below), enables faster, easier and better diagnosis of the state of virus populations, as a basis of therapy personalization. Campaigns for collaborative multi-stakeholder, interdisciplinary solutions for battling this infectious disease have also played an important role in contributing to the relatively fast impact on patient outcomes, although challenges remain, e.g. related to the high costs of many years of combination therapy close to the ‘cutting edge’ of molecular medicine. Interestingly, note that both areas (oncology and AIDS) have increasingly moved away from the use of single therapies, to a more sophisticated, cutting-edge combination therapy approach that involves the early recognition of disease recurrence. As a consequence, I propose a connected set of successful paradigms from oncology and ADIS as pillars of the platform described below, recognizing that much needs to be learned on how to apply those paradigms to chronic diseases with a more limited genetic contribution. In that context, a key question will be to find out where the most meaningful, interpretable and actionable diagnostic signals are, to guide our choice of interventions, based on the patient profile, at a particular stage in disease progression.

### Capturing the state of biological systems

Our ability to study, measure and understand complex biological systems has increased with many new tools and methods - although that doesn’t mean that it is easy to put the many different pieces of the puzzle together, in our mind, or in computational models. It is certainly more complex than ‘fixing a radio’ ( Lazebnik, 2002), although the author’s thoughtful points about unresolved issues in the biomedical research approach, including the lack of a formal language that helps communities to connect the pieces in such systems, were indeed very helpful, and influential. Enabling technologies in that area includes a maturation of our ability to capture states of biological systems at a more comprehensive level, using genome-wide technologies (or simply ‘ **omics’**). Such omics technologies now exist for many different levels of biological systems, including DNA, variants for RNA, protein and metabolite-level system dynamics (i.e. genomics, transcriptomics, proteomics, and metabolomics; Spivey, 2004). Depending on the sample we take and how we process it, omics technologies can generate very rich datasets about the ‘expression state’ of thousands of molecules in those systems (that are represented by the samples that were taken). However, there are many complex problems in data generation and interpretation as well. Inferring overall ‘health states’ (see below) from such measurements is possible, but non-trivial, and, at present, still resource intensive ( Chen *et al.*, 2012).

Around the year 2000, at about the same time as the hype on the sequencing of the human genome and its ability to revolutionize medicine, there was also much excitement on the promise of such omics technologies ( Spivey, 2004), leading to thousands of publications with datasets based on human and non-human samples (e.g. from species that are commonly used as preclinical models of human disease). However, most of those datasets represent ‘snapshots’ in time, with unclear positioning in terms of disease progression states, exact cellular composition, and other ‘metadata’ that would help with interpretation and comparison. Now that the first wave of excitement has given way to a second wave that aims to build on lessons learned from the first omics wave, there is increasing awareness about the importance of understanding disease progression, beyond ‘snapshots’ with limited ‘annotation’. This trend is likely to be enabling for the proposed approach, as it helps to connect ‘health states’ in time, with biology, at a comprehensive level. Looking back at how we handled omics waves could also be tremendously helpful in designing guiding principles for handling technology hypes in general.

### Engineering of patient-centric connected health solutions

Technical advances in a variety of areas, from mobile technology and the widespread use of smartphones, to health-related sensors, machine learning and digitalization of healthcare, are increasingly producing ‘real world’ impact based on convergence between different technology fields, beyond exciting prototypes, in chronic diseases ( Dobkin & Dorsch, 2011; Kvedar *et al.*, 2016). While the more difficult-to-change and highly regulated healthcare and health innovation sectors are expected to develop more slowly compared to less-regulated industries, e.g. those that can improve products quickly based on consumer-centric feedback loops, there are emerging paradigms with reusability potential. Patient-facing solutions with interfaces for other stakeholders, including healthcare providers, are one of the fastest-moving areas here.

For example, in respiratory diseases, such solutions have connected improved therapy (e.g. new COPD and asthma drugs) with ‘real world’ data on patient outcomes collected using mobile technology around ‘smart inhaler’ devices for those drugs, alongside with patient-centric views on smartphones, and the involvement of healthcare providers or clinical trial teams ( Bender *et al.*, 2017; Clift, 2016; Perez, 2015). This smart inhaler paradigm for designing “beyond the pill” solutions appears to provide value to multiple stakeholders, as a) the patients get better feedback on how they are doing with the inhaler-based therapy, including the aim to prevent stressful exacerbations, b) healthcare providers have more data to optimize care pathways, c) the developers of relevant drugs get more information on ‘real life’ settings and problems, enabling faster learning, while d) device developers get better feedback on how to optimize their devices in terms of usability, functionality and other health impacts, and how to connect the engineered systems with other components. Note that the ability to generate such value close to patients’ homes, outside classic healthcare settings (e.g. hospitals), is a factor driving excitement in the digital health sector, which has identified the management of chronic diseases as a key challenge and opportunity (for a more comprehensive overview, see Kvedar *et al.*, 2016).

In the context of the proposed platform (below), this and similar patient-centric paradigms fill an important void in the current healthcare and health innovation landscape, as they a) add low cost solutions closer to patients, in their natural environments, minimizing travel to clinics, b) have the potential to contribute diagnostic signals, and c) improve the ability to connect system components, across stakeholders, enabling a more data-driven approach to system-level learning.

### Measuring morbidity and disease burden, more globally

Improvements related to the direct and indirect effects of chronic disease morbidity (and improvements in terms of healthy aging) at population level need to be monitored in a reliable manner, to enable learning based on the impact achieved by different types of candidate solutions. The better we can measure impact, the more efficient our learning process. This needs to be based on a trusted methodology that works in a variety of settings, in different countries, to allow fair comparisons. Recent advances in this area include the “global burden of disease” (GBD) methodology developed by IHME (Institute for Health Metrics and Evaluation), and the framework developed by ICHOM, which establishes a first version of a system for capturing relevant impact. In terms of biomedical innovation, and the key role of clinical studies in validating specific hypotheses in human populations, we can now capture a diversity of patient outcomes, including QoL ( Guyatt *et al.*, 1993; Norman *et al.*, 2003) and health-related functions such as mobility (an emerging area enabled by sensors that record different kinds of movement patterns, e.g. accelerometry). At the economic sustainability level, measures such as QALY (QoL-adjusted life years) have added the highly debated ability to differentiate among years of life extension with high and low QoL when judging value provided by innovation ( Shiroiwa *et al.*, 2010). As a consequence, we now have a basic arsenal of tools to monitor the short and long-term results of solutions we develop, at different levels, in clinical trials, in regular clinical care as well as outside clinical settings. With this, we do have an improved ability to evaluate the impact of system-wide solutions that better connect the fragments, e.g. across the successful paradigms described in this article. However, this does not mean that the current toolbox for measuring outcomes across different settings is perfect and needs no further optimization. It is a very complex topic that will certainly require more innovation and adjustments down the road. At the same time, we can start to pragmatically use what we already have. In that context, multi-morbidity **,** in a landscape of increasing chronic disease burden, is one of the areas that may benefit from increased attention, with regards to the capture of impact, as well as the combination of several paradigms in relevant solutions.

### Open science culture

An intensive, open debate on the best approach for a particular problem, and open access to data that can help to select among alternative approaches, are features associated with *good science*. Since the successful paradigm of open source software in informatics has infected an increasing number of areas related to health innovation, including bioinformatics, the screening of chemical libraries, as well as the generation of tools for research on new kinds of drug targets, an intense debate has developed on the role for an extension of those fragmented experiences in particular areas of science into a more comprehensive, interdisciplinary ‘open science’ approach that includes innovative models for enabling a faster translation of research results to patients ( Laverty *et al.*, 2015; Low *et al.*, 2016; Munos, 2010). Indications in which innovative approaches at a similar level of complexity were tested ‘end-to-end’ have added further fuel to the debate, with the malaria field taking a prominent position among those translational pioneers ( Wells *et al.*, 2016). Relevant aspects include a) the openness of raw data, code and algorithms (avoiding ‘black box’ solutions), which applies to computational as well as experimental protocols, b) ‘reproducible research’ for enhanced transparency and reproducibility between research groups, c) sharing of data, insights, knowledge, and tools, based on initiatives that provide some structure to data sharing (e.g. *Dataverse*). Bernard Munos (e.g. Munos, 2010) has proposed that the increasing adoption of such approaches is linked with sustainability in health innovation, considering biomedical complexity. A recent conference in Oxford (“ Drug discovery: creating a new ecosytem”, 2–3 June 2016) has been able to gather a variety of pioneers in that area. However, while there are many experiences in this growing global community that can help to select a particular *open science* model for a given medical problem, we are still in the earlier stages of that learning process, in terms of tackling the rather difficult ‘valley of death’ problem in translation towards patients.

### Dealing with real world evidence

The digitalization of healthcare, as well as technology convergence in non-healthcare areas, are resulting in an increasingly diverse and fragmented landscape of data related to different aspects of health, from hospital records, to claims for reimbursement, to fitness device data, and data produced by patient-centric solutions for chronic diseases as outlined above ( Strategy & report, 2015). Such ‘real world evidence’ is often contrasted with data generated in controlled clinical studies, such as randomized clinical trials that test specific medical hypotheses. Important differences exist, for example, in our ability to make conclusions from either data, based on solid statistics, methodology and theory. In that context, it can be helpful to develop improved capabilities for dealing with *real world evidence* in a way that is consistent with ‘good science’ principles, in collaboration with disciplines that have a history in that area, e.g. epidemiology and HEOR (health economy and outcomes research). The example of the “Global Burden of Disease” study of IHME ( Lozano *et al.*, 2012), which succeeded in integrating thousands of different real world evidence data sources into an over-arching model that enables a number of analyses, could be helpful in that context.

### Business model innovation

Considering the important role of economy in the health care/innovation sustainability discussion (with an assumption of limited resources), we can observe that the classic economic model of reimbursement for healthcare actions based on a “fee-for-service” concept, is gradually being replaced by a potentially more sustainable “value-based care” model ( EFPIA, 2016). This model is based on the following principles: (i) coordinating around patients all the elements of the care continuum; (ii) shared commitment of all healthcare system players to the outcomes that matter to patients; (iii) generating and tracking data on those outcomes; (iv) benchmarking performance transparently for informing management decisions; and (v) paying for outcomes rather than for inputs and processes. However, this is a complex structural change and therefore unlikely to be an easy transition, so it may take decades before this transformation reaches all aspects of health-related systems globally (with progress being tracked by programs such as the Pharmaceutical Outcomes Research & Policy Program at University of Washington). In the meantime, pioneering institutions around the world are moving from pilots to organizational change, to become a leader in this important transition, increasing their fitness for the future at an earlier time point, when changes are still easier to manage and resource. One of the exciting opportunities in this area could be an improved ability to better align incentives across stakeholders, based on the above “value-based care” principles, as a basis for more collaborative solution development.

## Initial focus indications

### Alzheimer’s disease

Innovation related to this neurological disease is an interesting indication in the context of the proposed systems approach, and platform design, for several reasons:

- It is a common chronic disease with considerable burden on multiple stakeholders, including patients, families, healthcare resources, social care, elderly care, and other areas of society. This burden tends to rise in societies with longer life expectancies ( Braak & Del Tredici, 2015), and is therefore linked with aging populations.
- It features the typical slow and ‘silent’ disease progression of many chronic diseases (i.e. sporadic forms of the disease), as tissue damage accumulates over decades in the brain (e.g. as described by Braak & Del Tredici, 2015, at the level of pathohistology), with patient-specific speed of progression. There are known characteristics of subpopulations of patients with faster progression, e.g. based on genetic predisposition (presenilin families with early onset, APOE4 carriers with medium onset, others with late onset).
- Diagnostic complexity: experience with a very diverse collection of diagnostic tools, including those related to the detection of cognitive decline, molecular biomarkers, imaging biomarkers, histopathology. This could facilitate the iterative optimization of *health state* models (as proposed below).
- Culture of the field: Many years of disappointing results from clinical trials have resulted in a healthy ‘lessons learned’ discourse across disciplines, and stakeholders. A history of using computational modeling in the context of biomarkers for disease progression ( Haas *et al.*, 2016).

An interesting side effect of the history of disappointing or failed clinical trials in this indication is that it forces the biomedical research community to reconsider their approach and collaborative paradigms for the sake of patients, causing a healthy discussion on advancing the biomedical research community culture, with possible effects in other indications as well, where reusable learnings are generated. For example, this field has suffered from a strong bias for a small set of hypotheses and paradigms, based on particular types of evidence, as a basis for designing and developing therapeutic solutions. The excitement around those hypotheses has resulted in lackluster discussion of things that didn’t fit this ‘group think’, including alternative hypotheses and solutions. Compared to the discourse in this indication in the 1990s, we can now notice an increasing readiness to learn from this experience.

This neurological disease is a typical chronic disease in the pathobiology sense I discuss below, i.e. there is slowly accumulating tissue damage outpacing regenerative mechanisms, which results in a progressive decline of tissue functions, which then show up as increasingly severe clinical symptoms and patient outcomes are impacted over time. The need to better recognize early stages of disease ( Braak & Del Tredici, 2015), together with innovation in terms of interventions that target the pathobiology of exactly those stages, is now widely seen as the most promising approach for enabling translational progress in the field. This is likely to lead to sophisticated methods for combining diagnostic signals across many system levels, including the cognitive, molecular, imaging and other aspects described above, requiring a platform for improving public reference versions of relevant computational models.

At the same time, there has been good progress in areas related to digital health, e.g. in the early detection of a possible cognitive decline using speech patterns ( Morris & Lundell, 2003), which may add a cheap and easy-to-deploy screening method to the ‘early stage’ diagnostics innovation. The Alzheimer’s exemplar may also help in exploring connections between the various aspects of the proposed systems approach and platform. Below, I will propose a road map for developing such a platform, including starting points derived from Alzheimer’s disease.

Increasing readiness to design innovative clinical studies is also visible in this indication, related to particular subpopulations and health states, e.g. the APOE4 subpopulation, which carries a higher risk of fast progression towards more severe health states, compared to sporadic cases without such genetic risk factors ( Mahley *et al.*, 2009). This may help to close important gaps in the data landscape that prevent progress.

### Regenerative medicine, and chronic diseases

***Tissue and organ regeneration principles*.** We know that, based on knowledge accumulated in scientific fields related to *regenerative biology and medicine*, a) many animals have an amazing ability to regenerate tissues, organs and limbs (after injury or other damage), and therefore recover in terms of function (i.e. health); and b) that there is considerable evolutionary conservation at the level of the involved biology between humans and non-human vertebrate animals with high regenerative capacity. Discoveries in the past decades in that area have nurtured the hope that a deeper understanding of biomolecular systems involved in tissue, organ and limb regeneration will lead to the development of improved therapeutic and diagnostic solutions for areas of medical need, in which improved regeneration could contribute to better outcomes. In many common chronic diseases we can, during the progression of those diseases in the patient over time, observe a slowly progressing imbalance between accumulating tissue damage, and regenerative mechanisms activated in that tissue as a response to this accumulating damage. I will illustrate this principle using several examples, below, and, in the spirit of the proposed ‘systems approach’ to chronic diseases, highlight connected aspects from biology, medicine and economy that may enable the development of better solutions.

***Chronic liver disease*.** The liver is a very important organ, contributing to overall health with its many functions related to homeostasis (the ability to keep us within a healthy range, regarding physiological parameters). Depending on our lifestyle, and other factors, such as our genetic profile, liver tissue can be increasingly damaged by different factors, including high consumption levels of alcohol, and an unbalanced (Western) diet (leading to steatohepatitis). This then leads to reduced liver function, with an impact on our body’s ability to maintain homeostasis, and therefore health. On the other hand, the liver is also known for its regenerative capacity, a notion that was further reinforced by more recent observations of liver regeneration after the application of antiviral therapies ( Zois *et al.*, 2008). In the early stages of such slowly (and silently) accumulating organ damage, the liver may still be able to deal rather well with the repeated insults, and maintain most of the important functions, and therefore overall health. Over time, however, the accumulating damage outweighs the ability to regenerate and maintain organ function, shifting the system towards an unhealthier balance between damage and regeneration. As liver damage increases, and liver function decreases, first clinical symptoms may appear that are often ignored, for a variety of reasons. With a diagnosis of ‘late stage liver disease with advanced liver cirrhosis and portal hypertension’ by a relevant medical specialist (i.e. a hepatologist), a comprehensive reaction of the healthcare system (i.e. a care pathway) with diagnostic and therapeutic aspects is triggered, based on medical guidelines and current understanding of disease progression-related risks). While some patients’ lives can be extended through liver transplantation, those who die from liver disease on the transplantation waiting list, waiting for such relief, is unfortunately rising. This adds fuel to a discussion on the need for developing new solutions for this growing medical problem. What we know so far about the different stages of chronic liver disease in such patients can be summarized in the following simplified disease progression model, which includes a heterogeneous mix of causal factors involved in creating the liver tissue damage:

**

Here, each *health state* is a stage in disease progression that is characterized by a combination of features that can affect diagnosis, assigning a particular patient to an earlier or later stage in the disease progression (with many consequences related to the clinical management or further diagnostic monitoring of the patient). Based on current knowledge, it seems that, despite the heterogeneity of causal factors involved in creating the relevant tissue damage, there is a clear response pattern of this organ, with limited variation. In other words, once the balance between regeneration and damage has passed a certain level, a rather fixed pattern of progression towards later stages is observed. Some variation is noticed, however, between patients in the time spent between such ** stages, i.e. we can distinguish ‘fast’ or ‘slow’ progression relative to average times, between stages. As such chronic liver disease progression stages have multiple links with the overall health of the patient, in the context of the proposed *health state* modeling framework they can help to define health states, considering that the same patient may also display other co-morbidities, including other chronic diseases with their own progression stage.

In a similar manner, other chronic diseases result in slowly accumulating tissue damage over the years, until homeostasis and tissue function is affected to a degree that it becomes very difficult to get the patient back to a healthy state with a high QoL. Therefore, the liver disease example may help us define relevant paradigms for understanding *health states* that consider co-morbidity. Unfortunately, such accumulating tissue damage is currently not easy to detect in earlier stages, in real world settings that require a high standard of patient comfort, low cost, ease of use and risk mitigation. For example, repeated invasive sampling of biopsies for assessing the condition of liver tissue in the progression is usually not practiced due to clinical risks associated with biopsy generation. Therefore, we need to develop new solutions that will increase our ability to recognize not only health states and progression stages that have a clear clinical profile, based on currently available tools in the healthcare system, but also help us understand and recognize the health states that are in between very healthy states and the easier-to-recognize advanced stages of chronic diseases. This is a general principle and a grand challenge that no single discipline or stakeholder can fully address on their own.

With this context, what are some of the more interesting interdisciplinary connections involving biology, medicine and economics in developing such new solutions?

- **The NAFLD to NASH transition**: while many patients have NAFLD, i.e. a fat accumulation in liver that is not likely to be caused by excess consumption of alcohol, but rather by diet and other lifestyle factors (with interesting links to obesity and metabolic syndrome), only a subset of those patients are going to transition into the more serious NASH state in liver disease progression ( Calzadilla Bertot & Adams, 2016). In the NASH stage there is more serious tissue damage, possibly due to an overshooting reaction of the immune systems that is linked with the regenerative response to liver tissue damage. The transition from NAFLD to NASH is loaded with many questions, including the biology of this transition, the best ways to recognize it as early as possible in patients (e.g. Hannah & Harrison, 2016), and multiple economic consequences, such as the most efficient use of healthcare resources. While much progress has been made in understanding those aspects, a more collaborative approach across stakeholders is increasingly gathering momentum, including projects managed under the IMI umbrella (a framework for multi-stakeholder collaborations related to health, with public-private partnership at its core).
- **Effect of co-morbidities**: in patients with chronic liver disease, including earlier stages, what are the most relevant co-morbidities that will influence outcomes? While it is easy to see how factors that have a known effect on the regeneration/damage balance in the liver, e.g. exposure to substances with liver toxicity, will be relevant, epidemiological studies of ‘real world data’ collected on such patients may reveal additional factors that do not have a known liver tissue relevance. For example, such patients may take drugs (e.g. metformin) on a regular basis that are related to co-morbidities (e.g. diabetes), which interact in a complex manner with the liver disease progression system (e.g. liver functions related to glucose, He *et al.*, 2014). Statistically sound observations in such studies could then lead to investigations into the biology of those drugs in the context of liver disease, with potential effects on clinical management, outcomes and economy that would warrant interdisciplinary collaboration at such interfaces.
- **Refining the disease progression model**: While it is encouraging to know that there is a highly defined pattern in disease progression in liver diseases, despite the diversity of factors involved in causing tissue damage, there may be heterogeneity in patients that is currently under-appreciated. For example, if we consider the variability that was observed in terms of slow or fast progression between stages, including the risk of developing NASH based on NAFLD, this indicates that we need to learn more about patient heterogeneity and risk of progression, at the biology/medicine interface.

***Skin ulcers*.** The above discussion on chronic liver disease implies a fundamental challenge shared by other chronic diseases, i.e. that we are dealing with slow dynamics in the recognizable transitions between distinct *health states*. This means that our iterative learning cycle between designing a study, implementing it, sharing the results and designing the next study, combined with the time needed to observe sufficient change between health states, leads us to rather long studies that stretch many years. Therefore, fast learning based on short iterative cycles is difficult, apart from problems that can be addressed in shorter timescales. Together with an overall tendency towards short-term approaches including funding, this means that progress on understanding the above problems, including health state refinement, will be hard to accelerate.

With this in mind, let us explore the possibility of finding complementary medical problems related to a) similar interdisciplinary complexity of chronic diseases and b) learning related to health states, c) which would allow us to develop a fast-learning, collaborative network on top of short iterative study cycles and d) a systems approach that facilitates such interdisciplinary exchange. Our example for such a medical problem is again related to the principle of the fateful balance between slowly accumulating tissue damage, and scientific questions in the field of regenerative medicine. When our skin tissues are healthy and have a regenerative capacity within the normal range, we have all experienced how superficially visible wounds usually heal within a few weeks or even days. Once we look a bit closer at this area of tissue regeneration, we can notice a variation in terms of the speed and quality of healing, depending on a variety of factors, such as wound size, shape, depth, use of dressings to promote healing and prevent infection, infection management and so on. In addition, we may have heard about bad outcomes related to wound infection that led to amputations. Based on that common experience, most of us are not used to think of skin wounds as a major medical challenge in the context of the chronic disease challenge. However, if we look even closer, we find that there are many patients with one or more chronic diseases who have considerable problems due to disturbed healing, with surprisingly harsh outcomes linked with how wounds were managed ( Hunt *et al.*, 2011; Park *et al.*, 2013). But what is the link between the chronic disease challenge, and this medical problem? And how does it relate to our discussion on systems approaches?

**Diabetes complications**: Patients with diabetes, in later stages of disease progression, when slowly accumulating tissue damage has reached an advanced stage, may have to deal with a variety of clinical complications, affecting organs such as the eyes, kidney and foot. Complications of the foot typically present themselves clinically as ‘diabetic foot ulcer’, a type of non-healing, chronic skin wound, to a well-trained expert, such as a specialized wound nurse or physician ( Driver *et al.*, 2010; Lim *et al.*, 2017). Unfortunately, many patients carry such dangerous wounds for too long, and therefore have to face unfavorable outcomes, when it’s too late to manage the problem with currently available tools. Considering the progress we have made in terms of care coordination and clinical innovation in diabetes, this is one of the remaining problems related to diabetic complications.

**Other chronic diseases that affect skin regeneration**: To further complicate matters, other chronic diseases also have an effect on such tissue regeneration in the skin after wounding, including venous disease (i.e. leading to ‘venous leg ulcers’) ( Margolis *et al.*, 2002). Proper regeneration of the skin with a full restoration of tissue function (i.e. avoiding a scar with reduced function) requires many cells to do the right thing at the right time in the right context. Once the damage has occurred, there is a wave of signals going through the tissue that triggers that complex and dynamic regenerative response by many cells, including resident cells that go through all kinds of changes, as well as invading cells from the immune systems that arrive on the scene. As a result, a detailed molecular understanding of such skin regeneration is rather difficult, complicating efforts to develop new solutions based on that knowledge.

**Towards systems approaches**: I mentioned that many chronic diseases, such as liver disease, are difficult to study in terms of disease progression, because of the long timespans involved, which slow down the data-driven, iterative learning cycle. With regards to skin regeneration problems in the context of diabetes and other chronic diseases, the situation is a bit different, because a) changes related to outcomes can be measured in weeks and months, rather than years, b) the fluid produced by open wounds enables omics-type profiling close to the biology of tissue regeneration vs. damage, and c) the most affected tissue is relatively accessible, or easy to monitor, compared to tissues located further inside the body. The combination of those aspects could allow a fast-learning systems approach that combines the paradigms described above. This could then allow connections to be made between:

- **Biology** of ‘wound states’, e.g. improving our understanding of the balance between tissue regeneration and damage, how to shift it towards more regeneration, with potential benefits in other chronic diseases
- **Clinical** profile of ‘wound states’, e.g. when to intervene to prevent bad outcomes, and how to best integrate new diagnostics into care processes
- **Economic** profile of ‘wound states’, e.g. how to achieve good patient outcomes and economic efficiency, considering the secondary and tertiary effects of bad outcome wounds even outside the utilization of healthcare resources

Building a capability for fast learning based on short iterative cycles that enable data-driven approaches, including machine learning and expert-based learning, in the context of an interdisciplinary collaborative network, therefore seems an attractive opportunity in this area of medical need.

## Proposed platform

### Design principles guiding the development of the platform

I have mentioned the need to a) modernize diagnostics, extending the paradigms developed in leading areas of medical innovation, such as oncology and AIDS; b) connect better across system components that cross disciplines, e.g. medicine, biology and economics of health, e.g. in the context of more patient-centric *connected health* solutions; ** c) measure the impact of new PM solutions at that level, in a way that reflects the most relevant outcomes, enabling feedback loops that facilitate faster learning at systems level. But how can we best develop an enabling platform for such systems approaches based on those paradigms, which enables community-based learning, using open science principles, as well as feedback loops that involve real world evidence? Let us start with the center of the proposed platform, the *health states*, and their computational modeling across medicine, biology and economics.

Based on the knowledge we have accumulated in terms of disease progression in Alzheimer’s disease and chronic liver disease, I propose to aggregate the medical, biological and economic knowledge across these two indications in a way that allows the extraction of computational and theoretical platform components, as described below (with an eye on later reusability). In a next step, we would test the extension of such a two-indication platform to additional indications (including wound states reflecting skin regeneration), before we would explore the development of an even more comprehensive platform that captures all frequent chronic diseases, their progression stages and complexity at the level of multi-morbidity, for a particular patient. While such a comprehensive platform could have a variety of applications, its use in the design of sequential combinations of interventions, where timing depends on the recognition of a particular health state, is emphasized.

### Summary of enabling paradigms

- A modernization of diagnosis extending (and adapting) paradigms from oncology and AIDS, in parallel with innovation on interventions that build on diagnostic innovation
- Ability of omics profiling technologies to capture aspects of health states at genomic level
- Promoting the design of patient-centric *connected health* solutions based on *technology convergence* that provide value to multiple stakeholders, similar to the ‘smart inhaler’ paradigm
- Build on our increased ability to measure outcomes, morbidity and health at population level, to understand the impact of innovation
- Enabling faster community-based learning through a culture of sharing based on *open science*, FAIR and reproducible research principles
- Develop solid methodology for dealing with messy ‘ *real world’ data*, as a complement to more controlled clinical trial data, to understand the *patient journey*
- Facilitate business model innovation that aligns incentives across stakeholders, facilitating the economic success of more balanced, sustainable approaches
- Extend *systems approaches* capability through education and community building, see Figure 1 for scope and ambition (health state models may turn out to be only one component in the digital center between stakeholders, but nonetheless a pragmatic focus to learn how to better manage complexity)

### Stage 1: Building an initial platform, across two diseases

If we want to build an initial platform that captures current knowledge in both Alzheimer’s disease and chronic liver diseases, how could we get started, based on what exists already? In both indications, we have a relatively good understanding of disease progression states, from a medical, biological and economic point of view. This includes earlier stages of disease (when symptoms tend to be mild, with minimal impact on QoL and healthcare resource usage) to more advanced stages (when symptoms are more severe, with a more dramatic effect on QoL and healthcare resources). At the disease progression level, we can use the following starting points: a) Alzheimer’s disease progression theory of Braak & Del Tredici (2015); b) the review by Pellicoro *et al*. (2014), summarizing chronic liver disease progression. At the computational level, we can use the following starting points:

***Modeling health states*.** In Alzheimer’s disease, there is already a rich history of using computational modeling of distinct *health states* in the context of disease progression and disease severity, as reviewed by Green (2007). In the words of the author, “Markov models may be particularly useful when a decision problem involves clinical changes, across discrete health states, that are ongoing over time”, with such models “representing the course of a disease in terms of mutually exclusive ‘health states’ and the transitions among them”. For example, a 6-month cycle has been used to calculate transition probabilities between states, using clinical trial and epidemiological data. Such computational models are used to assess the value provided by medical interventions, e.g. those that result in a delay of progression towards severe health states. More recent updates on usage of such models, including additional applications, is provided by Green & Zhang (2016). Based on the small numbers of health states modeled so far, integration of additional data could lead to a population of alternative models with different numbers of health states, initially for Alzheimer’s disease only. It may also be necessary to extend the simplistic Markov modeling approach, e.g. considering progress made in projects like the 100K cohort ( Hood & Price, 2014) or the Google Baseline study ( Piller, 2015). Once an initial version of the computational platform is developed, it could be extended to chronic liver diseases.

***Semantic framework*.** IMI, a renowned multi-stakeholder platform for the development of **pre-competitive** assets with long-term effects related to medicine, has developed starting points in that area. This includes the Aetionomy project, which enables the representation of complex networks of information, including cause-and-effect relationships, based on the BEL language and the Semantic Web data format *RDF*. Braak and Del Tredici’s theory on connections between pathology and clinical symptoms in Alzheimer’s ( Braak & Del Tredici, 2015) can be generalized within the platform, in areas that are linked to the above health state models, to capture a slowly progressive tissue damage pathobiology, with regenerative biology context, affecting the dynamics of progression.

### Stage 2: Extend to skin regeneration

Therefore, the stage 1 platform would capture disease progression in two diseases, using Markov models from Alzheimer’s as a starting point, with knowledge from liver diseases, a very different indication with different characteristics, enabling the reusability-centric design of health state modeling and semantic framework. Although it would be desirable to then add new knowledge from both indications to the platform, their slowly progressing nature will limit the speed of such iterative optimization. To add faster-learning extensions that benefit the platform development effort, additional areas of medicine will therefore provide a natural focus of stage 2.

As discussed, skin regeneration as observed in non-healing wounds (skin ulcers) is a promising candidate for stage 2 medical focus areas. Based on existing knowledge on proteins found in wound fluid, a fluid similar to but also distinct from blood, panels of candidate protein-level biomarkers could be designed that capture ‘wound states’. Wound outcomes linked with such wound states and various ‘standard-of-care’ interventions could be tracked using nurse-centric *Digital health* tools that describe progress towards wound closure and healing (i.e. wound outcomes). Such tools could also capture features important for the early detection of potential complications, and facilitate the involvement of relevant experts based on wound states. Smart dressings for wounds that can benefit from negative pressure therapy are under development, within the Horizon 2020 program in Europe, potentially complementing the protein-based monitoring of wound states. In addition, new technologies are available for non-invasive assessments of skin architecture, based on confocal microscopy ( Lange-Asschenfeldt *et al.*, 2012). When connected into a comprehensive solution for tracking wounds in wound nurse type settings, such a *connected health* solution could not only generate valuable real world evidence on a diversity of ulcers, but also advance the ability to recognize and model health states as discussed above. For example, would the platform enable comparisons between innovation efforts in different countries and healthcare settings in a way that is more difficult at present? Testing their ability to model patient journeys in different populations, countries and healthcare settings could help to refine such models in a way that generates a second generation of reference models with an improved ability to capture ‘real world’ variation, where it matters. The more data become accessible for comparison and optimization, including new data types that extend our knowledge on the biology of different states, as well as diagnostic advances that change the health state recognition part of the models themselves, the more useful such health state models will become, in each iteration of improvement through data-based learning. Connecting them with tools early on that facilitate such iterative optimization will be crucial, including algorithms for the integration of multimodal diagnostic data related to such health states. Special attention should be given to make sure that different patient journey clusters are represented, if differences between those clusters can affect the definition or interpretation of health states, and transitions between health states.

***Additional indications*.** Beyond skin regeneration, additional areas of medicine that are currently not yet identified may become a focus in stage 2, if they present an opportunity to add faster-learning cycles to the health state learning process, within that platform. Candidate datasets include longitudinal observational human studies that generate data aimed at learning health states and state transitions, including those in early stages of disease, such as the 100K project ( Hood & Price, 2014) or the Google Baseline study ( Piller, 2015). Other reusable aspects of the platform may also require comparisons beyond those 3 initial indications, e.g. to design a reusable semantic framework around the health state modeling effort.

### Stage 3: A platform across many diseases

Once stage 2 is mature enough, the possibility of extension towards multi-morbidity in chronic disease progression space may present itself. Patterns of multi-morbidity frequently observed in epidemiological data could provide starting points for interdisciplinary focus, in terms of ‘real life’ health states composed of several chronic disease aspects. Limited resources in elderly care settings could provide an economic aspect, linked to an existing system of cross-indication progression monitoring, including tools for QoL and risk indicators in those settings.

At that stage, we may be able to approach a more generic and more patient-centric theory of chronic disease progression and health states, across indications. Convergence among the developments discussed above would facilitate its maturation. In particular, diagnostic innovation in slowly progressing chronic diseases would improve our ability to accurately diagnose different stages and variants of disease, including improved understanding of earlier stages of disease that are characterized by a clinically ‘silent’ progression of tissue damage that increasingly outpaces regenerative, damage control or repair mechanisms. In reference models that capture average disease progression in defined populations, transitions between the states in those models would be calculated using a variety of data, from different diseases, and different types of diagnostic evidence, to capture the characteristics of that population.

Once we have enough information about the most common health states and their reliable recognition, this new capability can be connected with machine learning capabilities that help to refine such models in various settings, based on an initial reference model and some starting conditions that, over time, increasingly fit the features of that particular setting (e.g. the educational and expectation profile of the involved participants, including healthcare providers and patients, as well as established processes, habits and culture). Optimization would be achieved based on feedback loops based on measured outcomes, including patient outcomes, as well as healthcare utilization and other economic aspects, at population level. Reference clusters of patient journeys could be refined, e.g. by adjusting the weights given to particular features in the clustering, filtering for the most predictive features, and including new features not covered in the reference model. Such adaptations should then find their way back to the next generation of reference models as well, so they become easier to adapt to different settings in the next round of optimization.

## Applications of the platform

In later phases of stage 2, and in stage 3, various applications of the platform can be envisioned. The examples below are meant for illustration, to encourage participation.

### Design of combined interventions

**Figure 2.**
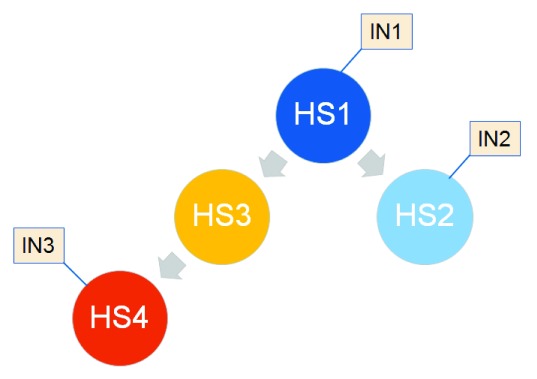
Design of combined interventions, as an application of health state modeling. Health states (HS1-4), which match state definitions in probabilistic Markov models, are connected with interventions (IN1-3), defining the time aspect in the PM vision (“the right intervention for the right patient, at the right time”). Each health state would have annotation in terms of pathobiology, health economics and clinical picture.

When single interventions are not sufficient to achieve the desired outcomes, combinations of interventions either delivered at the same time, or in a particular sequence over time (that considers increasingly refined health state recognition capability), will be an interesting option to consider in stage 3. This could mean that we will be increasingly able to iteratively approach a near-optimal personalized solution for a particular patient, at a particular time in their disease progression, extending the paradigms learned in oncology and AIDS to more indications, and into longitudinal data space. In principle, such value may not need to be limited to therapeutic interventions linked with health states, it could accommodate preventative interventions and even monitoring actions. For example, intervention ‘IN1’ designed for health state ‘HS1’ would lead to a subsequent health state HS2, which triggers intervention ‘IN2’, and so on. After ‘HS1’ there may be a branching point that, in some patients, leads to another state, ‘HS3’, which does not match well with intervention ‘IN2’, but requires monitoring that, once state ‘HS4’ is reached, triggers intervention ‘IN3’. Ideally, the diagnostic recognition of those states HS1-4 would be achieved with a single, reusable diagnostic procedure that is able to differentiate those health states based on non-invasive, low-risk approach ( Figure 2).

### Patient-facing solutions

Based on those reference models, user-friendly, patient-facing solutions could be developed, which compare the individual’s profile, at a particular time, with the most relevant reference model. If the individual’s profile is indicating faster-than-average progression towards one or more disease progression paths, a number of options could be explored, with tools that help monitoring the effect on progression at the level of health states. For example, the beneficial effects of lifestyle changes or therapeutic interventions on that profile could encourage continuation and compliance to relevant guidance. Gamification-related approaches could be useful in exploring aspects related to user engagement, considering expertise from user-centric design.

### Clinical studies

Similar usage may apply to clinical studies, if they span follow-up periods that contain *health state* transitions. In addition, increased ability to recognize distinct health states could generate hypotheses for new study tools linked with well-established measures and outcomes.

### Care coordination

Coordination of care, including the actions of different healthcare providers, as well as social/elderly care, is a very complex topic. The proposed platform could facilitate such efforts by simplifying the recognition of health states that require actions by specific components in the system, at a particular time, to then follow the effects of those actions on health state transitions more closely. For example, it could facilitate early recognition of worsening condition, complications, and other signals that require attention, and their collaborative interdisciplinary management.

### Care pathway redesign

While (health)care processes are often quite stable over time, once the team and process landscape is up and running, there are periods where such process landscapes are under discussion, to optimize particular outcomes, as well as economic constraints, e.g. for certain patient clusters that show high costs but below-average outcomes. Teams with a history of ‘care pathway redesign’ could start to engage in the proposed platform, as early as late phases of stage 2. If it is possible to improve the recognition of clinically and economically relevant *health states* in such settings, care pathway redesign projects would likely be able to extract value from such insights, as they try to determine the best time and mode to intervene in specific types of patient journeys. It could also help them with the exploration of a large range of options for care pathway changes, e.g. in a visual form that supports time-efficient discussion and consensus formation in complex, interdisciplinary groups, based on connected *health state* diagrams. On the other hand, such collaboration would enable early influence on the design of the proposed platform, to facilitate collaboration at such interfaces, which could become increasingly impactful on both sides over time as *health state* recognition (via diagnostic tools) and modeling add value to care pathway redesign projects. Such collaborations could therefore benefit both sides, the developers of the proposed platform, as well as the teams involved in care pathway redesign. This includes educational aspects required for such development, with the particular side effect that the platform community gets anchored into ‘real life’ settings as early as possible. Those focused on *disease biology* aspects of health states would benefit from such collaborations by improved anchoring of their efforts in ‘real life’ care settings as well.

### Device development

Institutions involved in device development could benefit from the platform in areas related to chronic disease progression, e.g. in projects aimed at developing smarter, more connected devices that contribute to multi-stakeholder value (similar to ‘smart inhalers’, see above). For devices linked with therapy application, the platform could help to manage projects, considering challenges related to the different timelines and cultures in therapy and device development, by allowing collaboration without excessive dependency between projects.

### Disease biology

Applications of the platform, related to disease biology, include:

- Enhanced ability to understand translatability of preclinical models, at the level of health state biology
- Improved ability to couple the development of novel therapies with biomarkers related to health states
- Gap analysis at portfolio level, using health states to aggregate project information

### Semantic web

By closely linking the effort on the development and optimization of *health state* models with initiatives focused on the representation of semantic aspects of relevant data, the following applications and value can be envisioned:

- Increasing adoption of semantic technologies, for the use of data in models
- Feedback on inconsistencies that help develop the semantic frameworks
- Further development of guiding principles (see below)

Recent progress made in relevant multi-stakeholder communities, such as FORCE11, towards consensus on guiding principles in related areas includes:

- FAIR Guiding Principles, to facilitate data and metadata re-use ( Wilkinson *et al.*, 2016)
- Increasing use of semantic web technology for many different types of biomedical data, e.g. RDF versions of EBI resources include diverse objects, from computational models to biosamples, chemicals and gene products ( Jupp *et al.*, 2014)
- Increased attention to the importance of capturing reusable metadata, close to data generation, in many institutions. While we are in early stages of connecting across such efforts, convergence with increasing consensus on how to apply FAIR principles will be a key challenge in the coming 5–10 years.
- Other developments that provide further fuel to such efforts are increasing awareness of the importance of a more consistent implementation of ‘reproducible research’ principles ( Walthemath & Wolkenhauer, 2016), to restore trust in the results of biomedical research

## A few obstacles to keep in mind

It is not for the lack of motivation, understanding or interest that systems approaches with similarity to the one discussed in this article have not developed towards real world impact, as measured by their contribution to the creation of tangible value to multiple stakeholders, and sustainability/health at systems level. Many obstacles have prevented or at least slowed down progress in that area, including the following factors, which deserve at least a brief discussion:

- **Project management** experience results in the reduction of complexity, prevention of scope creep and limiting the set of stakeholders involved in decision making, to **manage risks** associated with the ability to reach agreed deliverables, as well as stakeholder support. Such risk management also means that project leaders are forced to work with what exists, and need to often focus on value for particular stakeholders at the expense of others.
- A tendency to get infected by **technology hypes**, and other innovation fashions, which often lead to a shift in funding, attention and culture, which reminds us of a ‘gold rush’, including the ‘valley of tears’ after the hype, in which models fall apart, predictions are found to be wrong, credibility is lost, widespread frustration about unexpected complexity, and the failure of new wonder drugs ( Lazebnik, 2002). As Lazebnik put it, “this stage can be summarized by the **paradox** that the more facts we learn the less we understand the process we study”. If unmanaged, this very human tendency results in an inability to resolve problems at the systems level discussed in this article.
- Attitudes against **theory** development in science, the role of mathematics, and **computational** modeling as a tool, further complicate connections with some stakeholders. Such attitudes strongly depend on disciplinary background, highlighting the role of academic education and training in this phenomenon. Life science disciplines, such as biology and medicine, are well-known for a widespread disregard of those aspects, leading to unnecessary tensions with potential contributors from disciplines with stronger emphasis in those areas. An example is the history of omics technologies, where such attitudes and fixed mindsets from the days of a more reductionist “one postdoc, one gene” approach resulted in much waste of research resources due to a lack of experimental design, statistical analysis skills and theoretical background ( Micheel *et al.*, 2012). The way we approach problems, and hypes in particular, is at the root of the inability to advance in this area, as highlighted by Lazebnik (2002).

## Supplementary material

Supplementary File 1: Glossary of terms.
