# Supplementary material for: Towards a systems approach for chronic diseases, based on health state modeling
Source: F1000Res. 2017 Mar 23;6:309. [Version 1] doi: 10.12688/f1000research.11085.1 (PMC5419256; doi:10.12688/f1000research.11085.1)
Supplement: Supplementary file 1 [file f1000research-6-11956-s0000.tgz › 177dd6fa-2504-44e7-ac27-eb48e791cf7b.docx]

**Supplementary File 1: Glossary**

**Aging populations:** a shift in a country’s population towards older ages, i.e. a higher proportion of older people (above 60 years old) vs. younger people. A higher incidence of late stage chronic diseases that take decades to progress is a common consequence of aging populations.

**Big data:** in the context of this article, the ability to use many different kinds of data that are increasingly available on disease and related phenomena, due to increased digitalization, for applications related to health innovation. The above proposal is an example for designing interdisciplinary, collaborative frameworks which use computational modeling as an enabler to manage big data complexity in health.

**Biomarker:** is a measurable indicator of a biological state or condition, used to examine pathogenic processes or pharmacological responses to a therapeutic intervention. In other words, it is something of diagnostic value that can be measured in a quantitative and objective manner, including molecules, imaging readouts, histology stainings, and digital patterns (“digital biomarkers”) from smart devices. In the context of PM, and diagnostic modernization, biomarkers play an important role in the recognition of a particular health state.

**Diagnostics:** the process of determining by examination the nature and circumstances of a diseased condition, including the decision reached from such examination. It is the basis for decision making in the clinic, i.e. which intervention to use on which patient, at which time. This often means categorization in terms of disease, disease subtype, and progression stage.

**Digitalization:** integration of digital technologies into everyday life. In the above context, this includes the digitalization of healthcare, R&D and other areas related to health innovation, as well as data flow within and between those domains. In real life, there are many barriers to such data flow, creating ‘data silos’ e.g. within a hospital or R&D organization.

**Health State:** a recurrent pattern in human populations as they experience disease progression, which can be recognized based on the combined use of biomarkers, clinical and other data. At a simplified level, they represent stages of disease progression, as in Fig. 1 (liver disease). Due to increasing digitalization in health, we will become more granular and precise in diagnosis and health state recognition, as a basis for PM. See also Fig. 2, and the chapter on “modeling health states” (Proposed Platform). Developing a consensus representation of health states is one of the main goals of the proposed platform.

**Homeostasis:** is a system property in which a variable, e.g. body temperature, pH in blood, ion or concentrations in plasma, is actively regulated to remain very nearly constant. It is a concept from physiology, originally applied to a living organism’s body, including the human body. The ability of the human body to maintain homeostasis on a range of key variables is highly related to the above concept of health states, and disease progression.

**Disease progression:** the progression of a disease over time, e.g. from more subtle early signs of chronic disease that do not affect QoL substantially, to more advanced stages of disease with pronounced clinical symptoms and higher impact on QoL. For an example, see liver diseases (Fig. 1).

**Genetic stratification:** stratification based on DNA variants, a classic focus of the first wave of ‘Personalized Medicine’, which produced some successes in Oncology and rare diseases, but has more limited success in most chronic diseases (where DNA features capture only a subset of relevant signals for recognizing patient subpopulations with different needs in terms of care pathways). Therefore, PM visions such as those described in this article will require the development of new personalization paradigms beyond ‘genetic stratification’, for matching innovation at the level of diagnosis with interventions.

**Intervention:** an action taken by a healthcare provider, patient or another person involved in patient care (e.g. family members, social care) that has the potential to affect the health of the patient. This includes therapies (including but not limited to drugs) and preventive measures, as well as a decision to increase monitoring, or to change lifestyle.The proposed approach to health states couples a generic definition of interventions with diagnosis, monitoring and other aspects of health state recognition (e.g. in clinical trial situations).

**Longitudinal disease data:** health-related data gathered at multiple time points in the same patient(s), in a way that allows analysis of time-related phenomena, such as disease progression, and the delayed effect of interventions on health. Such data is the basis for understanding health state transitions.

**MCC:** the presence of multiple chronic conditions in the same patient, which can complicate healthcare.

**Open source:** an innovation paradigm that emphasizes the openness of algorithms and code in informatics, as a basis for progress made based on group learning in collaborative networks of contributors with relevant technical skills. This contrasts with ‘black box solutions’ that have a defined input and output, but hidden logic in between that is only accessible to some, e.g. within the institution that developed that solution. See also the discussion on Open Science above, as an extension of some Open Source concepts to other domains.

**QoL:** Health-related Quality of Life, a range of patient-centric measures that try to capture different aspects and consequences of morbidity, or health state changes, also in clinical studies. See Guyatt et al., 1993; Norman et al., 2003. Compare with QALY above.

**Paradigm:** a set of guiding concepts, theories and methods on which most members of the relevant community agree. In the history of science-based innovation, there are periods in time in which certain paradigms dominate, until a renewal process commences which results in a ‘paradigm shift’. Exemplars that illustrate a new paradigm in such a transition phase play an important role in synchronizing communication within a community as they debate new paradigms (Kaiser, 2012; [Kogan’s blog](http://koganbot.livejournal.com/109279.html))

**Real world evidence:** data that capture impact on health in ‘real world’ settings, outside the controlled, artificial environments that is characteristic for clinical trials that test specific hypotheses, typically in clinical centers with relevant capabilities for running such clinical studies. As they are defined by exclusion (of data gathered in clinical trials), they are by definition highly heterogeneous and not necessarily easy to use to test specific hypotheses. A great introduction is provided by Strategy& (2015). Note the overlap with digitalization and ‘big data’.

**Systems Approaches:** see Box 1.

**Technology convergence**: technology-related advances in more than one area form the basis for the engineering of engineered systems that deliver more health-related value than the individual advances. An example is the convergence between the use of smart inhalers for drugs against respiratory diseases like COPD and asthma, with sensors and mobile technology enabling the collection of data around the use of those inhalers by patients in home settings. Another example is the convergence of technologies that has enabled the first wave of Personalized Medicine in Oncology, based on ‘genetic stratification’, including a variety of technologies that enabled progress at the level of diagnosis and therapy, as well as the biology that is driving tumor behavior (see above).

**Valley of death:** the observation that a large number of promising preclinical insights with likely medical / health impact do not make it into validated medical tools that can be deployed in patient care, so they are accessible to patients who need it, in ‘real world’ settings. In a broader sense, it refers to the gap between the early stages of health-related innovation, and their translation into medical practice or patient-centric offerings. A good introduction into this important challenge for the new health innovation ecosystem is provided by Butler D (2008). Around the world, there is an intense debate on the need to redesign innovation systems in a way that they can better deal with challenges related to translation and the ‘valley of death’.
