# Supplementary material for: Towards a systems approach for chronic diseases, based on health state modeling
Source: F1000Res. 2017 Mar 23;6:309. [Version 1] doi: 10.12688/f1000research.11085.1 (PMC5419256; doi:10.12688/f1000research.11085.1)
Supplement: Peer review history

# Peer review: Referee response for version 1

**DOI:** 10.5256/f1000research.11956.r21818

**Published:** 2017-06-13

**Authors:** Charles Auffray, Bertrand De Meulder, Manlio Vinciguerra

**Affiliations:** 1 European Institute for Systems Biology & Medicine, Université de Lyon, Lyon, 69007 CEDEX 07, France; 2 European Institute for Systems Biology & Medicine, Université de Lyon, Lyon, 69366 CEDEX 07, France; 3 Center for Translational Medicine, International Clinical Research Center (FNUSA-ICRC), Brno, Czech Republic

This excellent paper proposes to learn from progresses in leading areas of medicine innovation (AIDS, oncology, Alzheimer's disease) to extend them to other chronic diseases using system approaches. Representing health states with the aim of designing the right intervention for the right patient at the right time and dose should allow reiterative approaches in longitudinal studies. The paper focus on modernization of diagnosis (omics etc), technologies/digital health and biological/clinical (biomarkers) and and economic aspects of the disease.

 A comment that can be done to the paper, only partially covered by other reviewers, is that it tries to cover a too large subject, which makes it difficult to read; while avoiding, in our opinion, some crucial points.

 It is also of note that the readability of the paper could be greatly improved by focusing on the key messages; the author tends to drift off topic and his lyrical musings, although interesting, add a lot of weight to an already large paper.

 We would omit/reduce the sections on Open science culture and Business model innovation, Semantic web.

 We would also reduce the section on systems approaches, that are already well covered in the manuscripts cited in the text.

Blue zones, islands of longevity: the author provocatively states how it is possible to translate the information that being a shepherd who eats barley and live in the mountain to the general population. Genetics of centenarians is a very important research field. We now know that specific mutations associated to IGF-1 are enriched only in centenarians. We can also learn a lot in terms of food habits and nutraceuticals from extreme long-lived healthy people. Diet, moderate intake or animal proteins. Exercise. Prevention.

 Family ties: Emma Morano Martinuzzi, the longest lived person on the planet recently passed away aged 117.5 years old. Emma had something that ALL long lived healthy people share: extensive family social network of support. A true army of offspring, grandchildren, cousins etc. Emma was never alone. In modern Western societies old people tend to isolate and die alone in their houses. 

 Loneliness is notoriously associated to lowering immune defenses, depression and chronic diseases. It is all linked.

An important section of the paper should be dedicated to governmental/social policies not to leave our elderly to age and die alone (robots are not a solution), which happens less in the Eastern world or in more archaic societies. 

 Medical knowledge, conceptual framework, technological advances and semantic categorization of stakeholders interactions are useless exercises without removing the elephant in the room: the sentimental and civil value we give to our elderly.

 Minor comment: in page 5, the author links the development of human civilization with the eutrophication of waters. We think that linking eutrophication with human activity should not be automatic. Eutrophication can be the product of a natural process, such as unusually high temperatures, modification of the hydrogeology of the ecosystem, natural erosion process of rich soils causing a sudden rise in availability of certain nutrients...

We have read this submission. We believe that we have an appropriate level of expertise to confirm that it is of an acceptable scientific standard, however we have significant reservations, as outlined above.

# Peer review: Referee response for version 1

**DOI:** 10.5256/f1000research.11956.r21819

**Published:** 2017-05-30

**Authors:** Jacques S. Beckmann

**Affiliations:** 1 Université de Lausanne, Lausanne, Switzerland

There is a general excitement about the perspectives of improved medical care and therapies afforded by the recent emergence of cost-effective high-throughput disruptive technologies, allowing the generation and computational analyses of massive amounts of clinically-relevant data and their transformation into useful information. This hype is evidenced by the abounding scientific literature on these subjects. The current manuscript summarizes the field approaching it from the perspective of pharma industries and focusing on chronic diseases.

 I read it more as representing as a first step, promoting a dialogue between the interested stakeholders, triggering reactions in order to eventually catalyse or crystalize the convergence of synergistic approaches.

 The manuscript lists numerous elements of discussion and consideration. This list is obviously incomplete, each reader may have his own favourite points to add. But this is also the purpose of this article (to which I could add my pet projects or thoughts).

 Indeed, in many aspects, this opening call to “put all the pieces of the puzzle together” may suffer from being yet incomplete, partially superficial or presenting an overly simplified interpretation; it may elicit disagreements on particular points. Yet, these limitations essentially reflect the uncertainties in which this field is currently navigating.

 As such the manuscript warrants publication in F1000, a forum where such discussions are encouraged.

 Specific comments:

 I like the wink to the tension between civilization, lifestyles and new technologies or what is referred to as “the other side of the coin”. Future discussions might also consider the potential risk of a further dramatic narrowing of the cultural, ethnic, species and environmental diversity, the consequences of which we don’t fathom fully as yet (although there have been numerous historical precedents, also illustrated in Y Harari's

 book).

 I would encourage further discussions on the deer necessity for the implementation of standardized, consistent nomenclatures and ontologies allowing, as suggested, cross-disease interactive channels. This is a real challenge as clinical data are much more complex and heterogeneous than lab data. To this we must add additional obstacles, when considering patient-centred involvement: these concern, among others, language (and cultural) barriers, the abundance of non-clinically approved monitoring devices and the heterogeneity in patients' verbal description of their status.

 Minor item:

 Correct ADIS to AIDS (under Modernization of diagnosis and personalized therapy)

I have read this submission. I believe that I have an appropriate level of expertise to confirm that it is of an acceptable scientific standard.

# Peer review: Referee response for version 1

**DOI:** 10.5256/f1000research.11956.r22492

**Published:** 2017-05-04

**Authors:** Rob Hooft van Huijsduijnen

**Affiliations:** 1 Independent consultant, Geneva, Switzerland

The author proposes to better exploit the vast and varied datasets that are being generated around  -specifically- chronic diseases to break these down in a set of 'health states' that can be used to guide improved prevention and treatment. Implementing such a view ('platform') requires the breakdown of barriers to Open Access and (paraphrasing) a revolution in health economics.

 These ideas are familiar to many who have spent time in the Pharma industry and sometimes lead to heated discussions. This reviewer, for one, subscribes to the majority of the views that are expressed here.

 The only real criticism I have is that the piece attempts to cover a lot of ground, and that the ideas could be presented in a more easily digestible way. Some very complex issues are lightly touched upon (the added Glossary is quite helpful). There are few specific, actionable recommendations- the way I read this is to prepare the mind-set for how better to tackle the great medical challenges of the 21 ^st^ Century. In itself that is OK if the message is clear.

 Some of the language is awkward- I would not focus on boring ' *health states*', for, in analogy with Tolstoy, ' *all happy families are alike*'. I guess what is actually meant here is ' *disease states*', and I would make that ' *disease stages* ' to convey the dynamics of healing and worsening disease.

 Overall, I had severe difficulties reading the title and abstract to make out what the article is about (dawned upon me much later). I initially half-expected this to be about holistic or anthroposophical medicine, or equivalent nonsense. In terms of informing and capturing the reader's attention I feel these sections can (and must) be much improved. The Journal *Nature* typically throws in 'Boxes' where complex concepts are explained. If the author is to reach a broad readership that includes academics this might be a good idea.

 The section of a changing medical landscape towards chronical diseases is interesting but the author does not specifically say why Pharma is inept to deal with these. The casual reader might ask here- "so what?" It is not clear how the 'Islands of healthy aging' fits in; I guess it is to say that genes and environment play important roles, and this analysis promotes the article's system approaches but then we are told that " *Other conclusions may be valid as well, and it can be difficult to choose among the alternative conclusions, to inform action*."- suggesting the problem is in fact intractable.

  Examples are given where modern systems biology has made good progress with improved diagnostics and interventions: oncology and AIDS. Fair enough, but these are diseases where the 'enemy' is crystal-clear. These disease states can almost be reduced simply to the metastatic tumour mass resp. virus load- it is not obvious the elegant solutions in this area are transferrable to 'regenerative medicine', the focus of the article. At some point it is said that ".. *biology of their [AIDS]  interactions with host (defense) biology, has enabled the development of highly personalized combination therapy approaches*". I disagree- almost all HIV drugs I know simply target viral enzymes or HIV's binding to CCR5. I just fail to see how these successes illustrate how we can tackle Alzheimer's, diabetes and similar diseases. The article would really gain from better suggesting how the proposed approach could help us in the right direction here.

 What I like however is the author's suggestion to be comprehensive in evaluating datasets that may affect the dynamics in disease stages. Also, his suggestion for better, global monitoring and standardized data collection merits follow-up. In this context the NHS could be cited, which is in a position where it can, and actually does such things. Also, I agree with the insight that we must let go of our ambition to fully understand systems. Some people understand how computers work, and some computers play better chess than any human being- likewise we will eventually understand the nuts and bolts of life- but remain unable to predict how humans will behave.

 I am a bit surprised that biomarkers are mainly presented as helping to understand 'disease states'. For me the main asset is that they can accelerate the evaluation of experimental drugs in patient (proxy outcomes). I am not too familiar with P4 and 'personalized medicine' for chronic diseases. I think the goal for now is to identify *any* treatment for, again, *e.g*., Alzheimer's for *any* category of patients- in my time we called that 'patient stratification'- but renaming elusive targets may provide some relief to frustration…

 Another topic that could easily be expanded into a doorstopper is combinatorial interventions. This reviewer fully agrees that the concept of treating complex diseases with a single molecule is outrageous. However, evaluating multiple combinations in a factorial clinical trial design is just impossible, to mention just one problem.

 The other topic that is (too) lightly touched upon is the disconnect between health care investment, outcomes and payback (health economics). I haven't seen much “value-based care”, with country-by-country (differential) pricing and too many clinical trials where NCEs are tested against placebos rather than the best existing care. Pharma (and Biotech) companies still behave as if they are just competing against each other, zero-sum-wise, spending more on marketing than on R&D in the process. As this article implies, they should rather be playing the game where they try to guess the cards that Mother Nature holds- and this requires cooperation. It is refreshing (and not wholly surprising) that such a proposal emerges from Novartis, one of the more enlightened Pharma Companies (no commercial interests with this reviewer).

 Many of the points raised above are intended to improve the important messages in this article and are to be seen as constructive criticism.

I have read this submission. I believe that I have an appropriate level of expertise to confirm that it is of an acceptable scientific standard.

# Response

**Published:** 2017-05-12

**Authors:** Michael Rebhan

**Affiliations:** Novartis Institutes for Biomedical Research, Switzerland

The referee has provided a very interesting, constructive, in-depth review of this article, identifying several aspects of the text that warrant the generation of a 2nd version of the paper with the aim of reduced ambiguity for specific aspects. As author my intention is to work with several co-authors who have relevant expertise on those improvements in the next months, so the community has a more useful reference text for a discussion of those aspects.

In the meantime, let me comment on some of his points right away, to further stimulate discussion:

1. Implementing the platform requires the breakdown of barriers to Open Access, and a revolution in health economics: Agreed, this is a tough challenge, for a variety of reasons - but what is the alternative we have as a society struggling with rapidly rising chronic disease burden, and healthcare costs? While it is clear that the current health economics in real life is quite different from the approach described in the paper, many pioneers increasingly agree that value-based models are an important part of a more sustainable approach we need to develop, and that fragmented discipline-specific innovation efforts will not get us there. Implementing it however is hard work, extremely complex, and likely an iterative learning process. It is not developing as fast as many of us would hope, on the ground, in many areas; but there is progress, e.g. look at the work of BD4BO in IMI, which helps to create an improved, practical foundation for such efforts. It is clear that we need to develop a more sustainable system, and that it is difficult to get fast traction in the real world, to a point where many of us may just give up once they know the true complexity of the challenge. But what is the alternative? Just keep talented innovators focused on easier-to-handle fragments with short-term effect that do not connect at system sustainability level? The vision of the paper may seem overly ambitious and idealistic to many short-term focused pragmatists, but I wonder if a bit more focus on long-term oriented, collaborative systems approaches such as what is proposed in this paper would create a more healthy balance in our health innovation ecosystem. Think of it as a portfolio approach to short and long term aspects, with the right balance between both.

See http://www.efpia.eu/topics/innovation/outcomes

2. There are few specific, actionable recommendations, it's more about preparing the mindset for learning how to better tackle the great medical challenges of the 21st century: I do not agree with this statement. Did the referee notice the proposed roadmap for developing a health state modeling platform, towards the end of the paper (stages 1-3)? The way it is described there it could be implemented with limited resources, in a few years, with some refinement along the way as the community builds, and data are collected on what works best. On the other hand, preparing mindsets to focus more on long-term sustainability may be a value nonetheless? 

3. Oncology and AIDS as examples: Partly agree. The referee comments that it is not obvious how the elegant solutions in those indications, where the root of the problem is more defined and easier to capture (e.g. at pathobiological level), can be transferred to chronic diseases and regenerative medicine. I agree that we cannot simply take the exact approaches that were used there, copy and paste them, and then apply them 'as is' in the indications described in the paper. As we accumulate more data on health states, learn how to represent and optimize such states, and build models for different uses, we need to learn which aspects and variants of those paradigms can be applied to which problems, and where the limitations are of a particular paradigm in terms of its application. There is no silver bullet that solves all problems, we all know that. Simply getting everyone's DNA and looking for signals there is not likely to be enough for chronic diseases with complex temporal change patterns, even if we do a lot of it in very large populations. Considering the proposed focus on the aggregation of longitudinal human data that can be simplified as health state models we need to first find out where the most relevant signals are that we should focus on.

4. Why is Pharma inept to deal with the changing medical landscape toward chronical diseases? This is an interesting question, which may best be addressed in a separate, follow-up paper, as it is quite complex. The intention of this paper is NOT to focus on a Pharma perspective. Instead, the intention is to zoom out and look at the problem from a more neutral but comprehensive perspective which is more likely to be relevant for different stakeholders, as described in Fig. 1. While the author cannot claim neutrality based on affiliation, a serious effort was made to avoid a Pharma bias in the text, and provide a more balanced perspective. The proposed platform for health state modeling can only work as an open crystallization point in the community if it achieves a healthy balance of interests (Fig. 1), but this certainly requires a transition from an atmosphere of blaming each other for failures at systems level, to true collaboration around more constructive and sustainable approaches. There are important fundamental problems that are hard to tackle, some of them mentioned by this referee, which require a new constructive culture that transcends institutions, interest groups and mindsets. Nobody said it will be easy. I think there are plenty of people in Pharma and other places who are ready to engage, not only in Novartis. Again, what is the alternative for society? Business as usual?

5. Biomarkers, health vs. disease states and patient stratification: The referee has many interesting points here, as the paper offers a somewhat unorthodox view of the role of biomarkers, in the context of patient stratification and disease progression. In the text, the way 'biomarkers' are discussed in terms of their relevance for the proposed health state modeling platform, as this is the focus of the text. Of course they will have additional uses and meaning, in other contexts. On the other hand, health states could also be defined in a way that not only relies on data related to biomarkers in a narrow sense, but also other objectively measurable signals that are usually not considered biomarkers. Once the health states have sufficient detail in terms of the most relevant disease biology states, linked with a clinical and economic profile, they could be seen as models that provide actionable interpretations and visualizations for combinations of biomarkers combined with other signals. The preference for the term 'health states' over 'disease states' comes from the idea that we still do not know enough about transitions between health and disease, and the earlier stages of many diseases. However, it is possible that therapeutic or preventative interventions in those earlier stages are likely to be more cost-effective and provide better patient value than later interventions. The way we look at disease and medicine currently is often focused on later stages with a strong phenotype that is visible in a classical clinical setting, with a higher hurdle for interventions to make a difference, and less emphasis on screening and preventative approaches. Again, this takes us back to economic reality in health, which may need a bit of adjustment, see above.

# Peer review: Referee response for version 1

**DOI:** 10.5256/f1000research.11956.r21975

**Published:** 2017-04-24

**Authors:** Michel Goldman

**Affiliations:** 1 Institute for Interdisciplinary Innovation in Healthcare, Université Libre de Bruxelles, Brussels, Belgium

Excellent article that will be inspirational for shaping the future of medicine and healthcare. The systems approach should indeed revolutionise the approach of chronic disorders. The authors rightly integrate the importance of patient-centricity and digital health to translate precision medicine into standard of care. The F1000Research Open Science is a very appropriate vehicle for dissemination of this innovative vision.

I have read this submission. I believe that I have an appropriate level of expertise to confirm that it is of an acceptable scientific standard.
